# Patterns of complication burden in people with diabetes: a latent class analysis approach

**DOI:** 10.1007/s00592-026-02703-7

**Published:** 2026-05-06

**Authors:** Bárbara Maria Farias Kruschewsky, Roseanne Montargil Rocha, Marcelo Araújo, Rafael Ernane Andrade, Icaro J. S. Ribeiro

**Affiliations:** 1 Health Science Department, Graduate Program in Nursing, State University of Santa Cruz, Campus Soane Nazaré de Andrade, Road Jorge Amado, km 16, Salobrinho, Ilheus, Bahia 45662-900 Brazil; 2 Health Science Department, State University of Santa Cruz, Ilheus, Bahia Brazil; 3 Beira Rio Hospital, Itabuna, Bahia Brazil

**Keywords:** Diabetes mellitus, Risk stratification, Latent class analysis

## Abstract

**Aims:**

To identify patterns of complication burden among individuals with diabetes mellitus based on sociodemographic, behavioral, and clinical characteristics, and to examine their co-occurrence with diabetes-related comorbidities.

**Methods:**

This cross-sectional study was conducted during a diabetes health campaign in a municipality in southern Bahia, Brazil, involving 1,542 patients. Data were obtained through a standardized questionnaire and ophthalmological examinations. Latent class analysis was applied to identify subgroups with similar clinical characteristics. Models with two to four classes were estimated, with the two-class model presenting the most parsimonious and interpretable solution according to BIC. Associations between classes and comorbidities were estimated using Poisson regression with robust variance, adjusted for age and sex.

**Results:**

Two classes were identified. Class 1 (86.6%) showed lower complication burden, with preserved vascular and sensory function. Class 2 (13.4%) was characterized by a higher frequency of ulceration, amputation, absent peripheral pulses, and impaired protective sensation. Individuals in Class 2 presented higher prevalence of cardiovascular disease (PR = 1.47), myocardial infarction (PR = 1.64), neurological disease (PR = 1.67), and retinopathy (PR = 1.63).

**Conclusion:**

The identified classes primarily reflect differences in peripheral complication burden, with higher co-occurrence of vascular and microvascular conditions in the more affected group. These findings describe patterns of complication clustering within a screening population and may support population-level strategies for identifying individuals with greater healthcare needs.

## Introduction

Diabetes mellitus (DM) is one of the leading noncommunicable chronic diseases and represents a growing challenge for public health worldwide. It is estimated that approximately 537 million adults aged 20 to 79 years are living with the disease globally, a figure that may reach 783 million by 2045 due to population aging and changes in lifestyle patterns [1]. In Brazil, the crude prevalence of diagnosed diabetes in 2019 was 7.7% (7.4–8.0), representing a relative increase of 24% compared with the prevalence observed in 2013 [2].

In 2023, approximately 30 million diabetes-related visits were recorded in primary health care units across the country, highlighting the high demand for continuous and specialized care [3]. The management of DM is complex due to the presence of multiple associated comorbidities. Notably, cardiovascular diseases (CVD) [4], myocardial infarction (MI) [5] and stroke (AVC) [6], stand out as the leading causes of mortality in this population [7].

In addition, microvascular complications such as diabetic retinopathy, nephropathy, and peripheral neuropathy are prevalent and have a substantial impact on patients’ quality of life [8–10]. Furthermore, the coexistence of arterial hypertension, dyslipidemia, and obesity exacerbates cardiovascular and metabolic risk, requiring integrated and multidisciplinary approaches [11, 12].

Thus, beyond its high prevalence, DM exhibits substantial clinical, genetic, and socioeconomic heterogeneity, affecting populations with diverse risk profiles, disease trajectories, and therapeutic responses [13, 14]. This underscores the need to develop specific patterns of complication burden across population subgroups [15, 16]. Accurate stratification of these profiles is essential to inform clinical decision-making contributing to hypothesis generation for future multidimensional phenotyping approaches. Within this context, investigating the population groups most affected by DM is imperative.

Accordingly, the present study aims to identify patterns of complication burden among individuals with diabetes mellitus based on sociodemographic, behavioral, and clinical characteristics, and to examine their co-occurrence with diabetes-related comorbidities.

## Methods

### Study design

This is a cross-sectional study conducted during a Diabetes Health Campaign in a city in southern Bahia, aimed at providing multidisciplinary care to patients with diabetes affiliated with the Brazilian Unified Health System (SUS).

### Participants and data collection

Individuals with a prior diagnosis of DM, aged 18 years or older, residing in the city where the campaign was conducted and receiving care through the Unified Health System (SUS) were included. Participant recruitment was conducted through convenience sampling, including direct invitation by researchers and dissemination via media. Therefore, the sample reflects individuals voluntarily attending a health campaign and does not represent a population-based sampling frame. As a convenience sample, it may overrepresent individuals who are more health-conscious, symptomatic, or with more advanced disease, limiting the generalizability of the findings.

Data collection was performed by medical and nursing students who were appropriately trained and supervised by health professionals. Participants completed standardized clinical and epidemiological questionnaires. In addition, they underwent ophthalmologic examination, including pupil dilation and retinography, for detailed retinal assessment.

Pre-analytical variables were also evaluated, including fundoscopic examination results (presence or absence of retinographic abnormalities), diabetic retinopathy (present or absent), presence of established diabetic nephropathy, medical history, smoking status, medication use (insulin, oral antidiabetic agents, or other medications). Neurological disease was defined based on self-reported medical diagnosis of neurological conditions (excluding diabetic peripheral neuropathy, which was assessed separately through protective sensation testing). Clinical evaluation included assessment of ulceration, amputation, posterior tibial (PT) and dorsalis pedis (DP) pulses, and protective sensation.

### Statistical analysis

To identify clusters of patients with similar characteristics, latent class analysis (LCA) was applied. The included indicators were selected based on clinical relevance and their potential to distinguish patterns of complication burden within the screened population. In LCA, variables should meaningfully contribute to class separation rather than introduce redundant information; therefore, indicator selection prioritized constructs that captured complementary aspects of peripheral impairment [17].

Models specifying one to four latent classes were estimated using maximum likelihood via the expectation–maximization (EM) algorithm. Model selection followed principles of parsimony, statistical adequacy, and substantive interpretability [18]. Fit was evaluated using log-likelihood values, Akaike Information Criterion (AIC), Bayesian Information Criterion (BIC), likelihood-based goodness-of-fit statistic (G^2^), and entropy. Because AIC applies a lighter penalty for model complexity and may favor overparameterized solutions, greater weight was given to BIC for class enumeration. Lower values of AIC, BIC, and G^2^ were interpreted as indicating improved fit after accounting for model complexity. Entropy was examined as a measure of classification precision but was not used as the primary determinant of the number of classes [19].The average posterior probabilities (AvePP) for class membership were examined to assess classification quality. High mean posterior probabilities indicate adequate separation between classes and support the reliability of modal assignment for descriptive purposes.

To formally compare nested solutions, the Vuong–Lo–Mendell–Rubin (VLMR) and Lo–Mendell–Rubin adjusted likelihood ratio tests were conducted. The comparison between the one- and two-class models was statistically significant (p < 0.001), indicating that a homogeneous (single-class) model was insufficient to represent the data structure. Although the two- versus three-class comparison also reached statistical significance (p < 0.01), the three-class solution generated a very small class (2.1% of the sample) and yielded a higher BIC compared with the two-class model. Considering parsimony, convergence stability, and interpretability, the two-class solution was retained for subsequent analyses.

All models were estimated in R (version 4.5.0) using the *poLCA* package. To reduce the risk of local maxima, each model was estimated using multiple random starting values (n = 50 replications), and convergence was verified through replication of the maximum log-likelihood across runs.

Individuals were assigned to latent classes based on the highest posterior probability (modal assignment). Subsequently, Prevalence ratios (PR) and 95% confidence intervals were estimated using Poisson regression models with robust variance estimators, adjusted for age and sex, given the cross-sectional design and the non-rare frequency of outcomes. Analyses were conducted in R (packages *poLCA*, *ggplot2*, and *cowplot*) and Stata (version 12).

### Ethical aspects

As this study involved human participants, the ethical standards established by Resolution No. 466/12 and its complementary guidelines were fully observed. The project to which these data are linked, entitled “Evaluation of the main complications observed in diabetic patients treated at the Diabetes Health Campaign in Itabuna, Bahia,” was duly submitted to and approved by the Research Ethics Committee, under Opinion No. 5779758/2022.

## Results

A total of 1,542 individuals were evaluated, with a mean age of approximately 62 years (± 11.76), ranging from 18 to 101 years. Females predominated (64.66%), and most participants had completed elementary education (50.48%). Regarding clinical characteristics, the mean duration of DM was approximately 10 years, and 21.89% of participants used insulin. To examine different patterns of complication burden, three latent class models were constructed with two, three, and four classes (Table 1).

**Table 1:** Fit indices of the tested latent class models

Number of classes	LLIK	BIC	AIC	G2	Entropy
2	**− 7080.13**	**14428.12**	**14242.55**	620.99	0.818
3	**− **7100.28	14505.28	14254.26	635.42	**0.870**
4	**− **7060.66	14583.79	14247.32	**604.44**	0.794

*AIC* Akaike Information Criterion, *BIC* Bayesian Information Criterion (BIC), *G*^*2*^ likelihood ratio test statistic; *LLIK* log-likelihood

Model selection considered AIC, BIC, G^2^, log-likelihood, and entropy. Although the 4-class model presented lower AIC and G^2^ values and the 3-class model showed higher entropy, the 2-class model yielded the lowest BIC. In latent class modeling, criteria that impose stronger penalties for model complexity, such as BIC, are often preferred to reduce the risk of overfitting and to favor more parsimonious and stable solutions. Moreover, model selection in LCA should balance statistical fit with substantive interpretability and theoretical coherence. Additionally, although the VLMR and LMR tests indicated statistical improvement for the 3-class solution (p < 0.01), the additional class comprised only 2.1% of the sample and did not substantially improve BIC compared to the 2-class model. Given the small class size and the higher BIC value, the 2-class solution was retained based on parsimony and stability considerations.

The mean posterior probabilities for class membership were high for both classes (Class 1 (AvePP = 0.85); Class 2 (AvePP = 0.91), indicating good classification precision and clear separation between latent profiles.

Thus, the two classes consisted of three sociodemographic indicators (i.e., age, sex, and educational level), three lifestyle indicators (i.e., alcohol consumption, smoking, and physical activity), and five indicators related to foot health assessment (i.e., ulceration; amputation; PT and DP pulses; and protective sensation).

Class 1 comprised 86.6% of the evaluated individuals and showed higher probabilities of including those aged 60 years or older (0.62), female (0.65), with incomplete or complete elementary education (0.51), non–alcohol consumers (0.86) and non-smokers (0.94), physically active (0.84), without ulcers (0.98) or amputations (0.99), with PT (0.95) and DP pulses (0.98), and preserved protective sensation (0.82). Class 2 represented 13.4% of the individuals and followed a pattern similar to that of Class 1; however, it presented higher probabilities of smoking (0.08), presence of ulcers (0.09) or amputations (0.05), absent PT (0.66) and DP pulses (0.42), and impaired protective sensation (0.25) (Table 2).

**Table 2:** Prevalence and latent class probabilities of clinical profiles among individuals with diabetes

	Class 1	Class 2
Class prevalences	1335 (86.6%)	207 (13.4%)
Indicators		
*Age*		
< 60 years	0.38	0.44
≥ 60 years	0.62	0.56
*Sex*		
Female	0.65	0.63
Male	0.35	0.37
*Educational level*		
Illiterate	0.15	0.14
Incomplete/complete elementary	0.51	0.56
Incomplete/complete secondary	0.28	0.27
Incomplete/complete higher	0.06	0.03
*Alcohol consumption*		
No	0.86	0.94
Yes	0.14	0.06
*Smoking*		
No	0.94	0.92
Yes	0.06	0.08
*Physical activity*		
No	0.16	0.08
Yes	0.84	0.92
*Ulcer*		
No	0.98	0.91
Yes	0.02	0.09
*Amputation*		
No	0.99	0.95
Yes	0.01	0.05
*Posterior tibial pulse*		
Palpable	0.95	0.17
Unilateral	0.04	0.17
Absent	0.01	0.66
*Dorsalis pedis pulse*		
Palpable	0.98	0.41
Unilateral	0.02	0.17
Absent	0.00	0.42
*Protective sensation*		
Preserved	0.82	0.75
Absent	0.16	0.25

Itabuna, Bahia, 2025

Given the increased prevalence of certain indicators in Class 2, this group was identified as having a higher complication burden in the current study. Accordingly, associated pathologies were examined, individuals in Class 2 exhibited a higher co-occurrence of cardiovascular diseases (PR = 1.47; [95% CI 1.09–1.97]), myocardial infarction (PR = 1.61; [95% CI 1.11–2.34]), neurological disease (PR = 1.63; [95% CI 1.03–2.61]), and retinopathy (PR = 1.65; [95% CI 1.27–2.15]) (Table 3).

**Table 3:** Prevalence ratios (PR) and 95% confidence intervals (95% CI) of the evaluated pathologies according to latent class (1 or 2), adjusted for age (< 60 or ≥ 60) and sex

	Class 1		Class 2		PR (95%CI)^1^
	n	%	n	%	
*Hypertension*					
No	401	30.61	54	26.09	1
Yes	909	69.39	153	73.91	1.21 (0.90–1.62)
*Obesity*					
No	1203	91.83	191	92.37	1
Yes	107	8.17	16	7.73	0.97 (0.60–1.57)
*Cardiovascular disease*					
No	1104	84.27	160	77.29	1
Yes	206	15.73	47	22.71	1.47 (1.09–1.98)*
*Myocardial infarction*					
No	1214	92.81	182	87.92	1
Yes	94	7.19	25	12.08	1.64 (1.13–2.39)*
*Chronic kidney disease*					
No	1265	96.64	197	95.17	1
Yes	44	3.36	10	4.83	1.34 (0.75–2.40)
*Neurological disease*					
No	1256	95.88	192	92.75	1
Yes	54	4.12	15	7.25	1.67 (1.04–2.68)*
*Retinopathy*					
No	1027	78.40	138	66.67	1
Yes	283	21.60	69	33.33	1.63 (1.25–2.12)*

Itabuna, Bahia, 2025

^1^Estimated using Poisson regression with robust variance, adjusted for age and sex.Adjusted for age (< 60 and ≥ 60) and sex *Statistically significant difference

## Discussion

This study identified two latent classes among individuals with diabetes, reflecting distinct levels of complication burden within a screening population. The class with a higher burden was primarily characterized by indicators of peripheral vascular and neuropathic impairment, including ulceration, amputation, absent posterior tibial and dorsalis pedis pulses, and impaired protective sensation. Individuals in this class also showed a higher prevalence of cardiovascular disease, myocardial infarction, neurological disease, and retinopathy. In contrast, the lower-burden class showed a predominance of preserved vascular and sensory function.

These findings indicate that the identified classes primarily represent a gradient of peripheral complication burden rather than comprehensive multidimensional clinical phenotypes. The structure of the model was largely driven by foot-related clinical indicators, which capture advanced manifestations of vascular and neuropathic impairment, reflecting heterogeneity in complication burden among individuals with diabetes [20]. In this context, its contribution lies in describing how complication burden is organized, rather than redefining established clinical categories or supporting individual-level clinical decisions [21, 22]. This approach may contribute to resource allocation and population-level planning, particularly in complex chronic conditions such as DM [23, 24]. However, its applicability at the individual level remains to be established.

The higher complication burden class showed a greater co-occurrence of cardiovascular disease, myocardial infarction, neurological disease, and retinopathy. Given the overlap between class-defining indicators and these outcomes, particularly in relation to vascular impairment, these associations are clinically expected and should be interpreted as patterns of co-occurrence rather than independent relationships. Considering the cross-sectional design, these findings do not imply causal or prognostic associations, but instead reflect the simultaneous presence of multiple complications in individuals with more advanced disease [25, 26].

From a population health perspective, the identification of a subgroup with a higher burden of complications within a campaign-based screening context contributes to understanding how complex clinical manifestations aggregate in real-world settings. These findings are descriptive and should be interpreted as complementary to existing clinical frameworks, without direct implications for individualized therapeutic decision-making.

The co-occurrence of vascular, neuropathic, and microvascular complications observed in the higher burden class is consistent with the known clustering of advanced diabetes-related complications, with an increased risk of plantar ulcers, infections, and nontraumatic amputations [27–31].

Some limitations should be considered. The cross-sectional design precludes establishing temporal relationships between class-defining indicators and associated comorbidities. The higher burden class likely represents individuals with more advanced or cumulative disease manifestations, rather than a group at increased prospective risk. Additionally, the classes were influenced by peripheral complication indicators, particularly foot-related variables, and did not include key metabolic parameters such as glycemic control, lipid profile, or blood pressure. This limits interpretation as a comprehensive cardiometabolic phenotype. Finally, class assignment was based on modal posterior probabilities without three-step correction, which may introduce classification error and potential bias in associations with external variables [32].

Future research should evaluate the generalizability of these patterns across diverse populations and incorporate longitudinal designs and broader clinical indicators to assess their stability and potential relevance in different contexts.
